# Disinfection

**Published:** 1897-05-15

**Authors:** 


### DISINFECTION.

In a paper on the subject of Disinfection, read before the members of the Civil and Mechanical Engineers' Society, on April 22nd, Mr. W. N. Twelvetrees gave a careful description of the various forms of apparatus in use for disinfecting purposes.

Speaking of superheated and saturated steam, he said there was an important difference between them. Experiment had shown that superheated steam at 285 deg. Fahr. acting for 22 minutes on spores of bacillus anthracis had no sterilising effect, whereas the action of saturated steam at 213·80 deg. Fahr. for eight minutes entirely killed a portion of the same cultivation.

Saturated steam, when introduced to the interior of a disinfecting chamber, condensed so soon as it met any objects which were of lower temperature. The steam in condensing became a thin layer of water, giving up latent heat, which was absorbed by the objects. More steam then occupied the space left vacant by condensation, and the same operation was repeated until the whole of the objects were raised to the temperature of the steam, and penetration was complete. This result was usually arrived at within 15 minutes, the time depending, of course, on the thickness of the material to be penetrated.

Superheated steam, on the other hand, being a perfect gas, acted simply by conduction, and was even slower in its action than hot air. The only useful manner in which superheated steam might be employed was for drying purposes after disinfection had taken place. If so used, care must be taken to prevent superheating at too early a stage.

